# Three-Dimensional Culture System of Cancer Cells Combined with Biomaterials for Drug Screening

**DOI:** 10.3390/cancers12102754

**Published:** 2020-09-24

**Authors:** Teruki Nii, Kimiko Makino, Yasuhiko Tabata

**Affiliations:** 1 Laboratory of Biomaterials, Institute for Frontier Life and Medical Sciences, Kyoto University, 53 Kawara-cho Shogoin, Sakyo-ku, Kyoto 606-8507, Japan; nii.teruki.85x@st.kyoto-u.ac.jp or; 2 Faculty of Pharmaceutical Sciences, Tokyo University of Science, 2641 Yamazaki, Noda, Chiba 278-8510, Japan; makino@rs.noda.tus.ac.jp; 3 Center for Drug Delivery Research, Tokyo University of Science, 2641 Yamazaki, Noda, Chiba 278-8510, Japan

**Keywords:** biomaterials, tissue engineering, 3D cell culture, cancer cells

## Abstract

**Simple Summary:**

For the research and development of drug discovery, it is of prime importance to construct the three-dimensional (3D) tissue models in vitro. To this end, the enhancement design of cell function and activity by making use of biomaterials is essential. In this review, 3D culture systems of cancer cells combined with several biomaterials for anticancer drug screening are introduced.

**Abstract:**

Anticancer drug screening is one of the most important research and development processes to develop new drugs for cancer treatment. However, there is a problem resulting in gaps between the in vitro drug screening and preclinical or clinical study. This is mainly because the condition of cancer cell culture is quite different from that in vivo. As a trial to mimic the in vivo cancer environment, there has been some research on a three-dimensional (3D) culture system by making use of biomaterials. The 3D culture technologies enable us to give cancer cells an in vitro environment close to the in vivo condition. Cancer cells modified to replicate the in vivo cancer environment will promote the biological research or drug discovery of cancers. This review introduces the in vitro research of 3D cell culture systems with biomaterials in addition to a brief summary of the cancer environment.

## 1. Introduction

**Figure 1:**
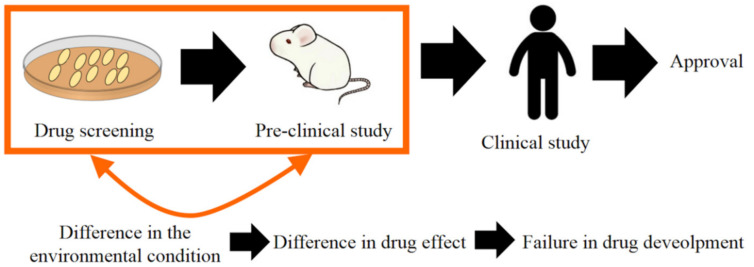
Research and development process of drug development. The difference in the environment condition between in vitro and in vivo leads to that in drug effects, which often causes a failure in drug development.

The basic concept of regenerative medicine is to achieve the regeneration and repairing of damaged or injured tissues by utilizing the natural healing potential of the body itself. Regenerative medicine consists of regenerative therapy and regenerative research. Regenerative therapy is to treat patients through the in vivo enhancement of cell activity. Regenerative research is positioned as the scientific support for the regeneration therapy of the next generation. Drug discovery is defined as regenerative research. The therapeutic efficacy, metabolism or toxicology of drugs are efficiently evaluated by taking advantage of activated cells. To enhance the cell activity, two methodologies have been recently noted. One is to utilize three-dimensional (3D) cell culture technologies. Cells are usually cultured in a two-dimensional (2D) system, with a plate or dish. However, the functions of cells cultured in the 2D system are lower than those of body cells because cells tend to interact with each other for the enhancement of their own activities in the body [1,2,3,4]. Due to the difference in the cell condition, the drug effect evaluated by the in vitro drug screening is not always the same as that in preclinical or clinical study, which leads to the failure of drug research and development [5,6] (Figure 1).

The comparison of cancer cell culture between 2D and 3D systems is shown in Table 1. There are merits or demerits between the two culture systems. Although the systems have been used depending on the purpose, the 3D culture is superior in terms of drug discovery which well reflects the in vivo cancer environment. The other methodology to enhance cell functions is the active utilization of biomaterials. Cell culture is often performed on the dish or plate which is mainly composed of polystyrene. This condition of an artificial environment is quite different from the in vivo body environment of cancer cells, and consequently, the drug effect or cytotoxicity evaluation is technologically limited. Biomaterials which consist of extracellular matrix (ECM) components are effective in enhancing the cell activity or functions. The interaction with biomaterials will enable cells to enhance their proliferation, differentiation, and biological functions, leading to the realization of cancer cell–environment interaction.

**Figure 2:**
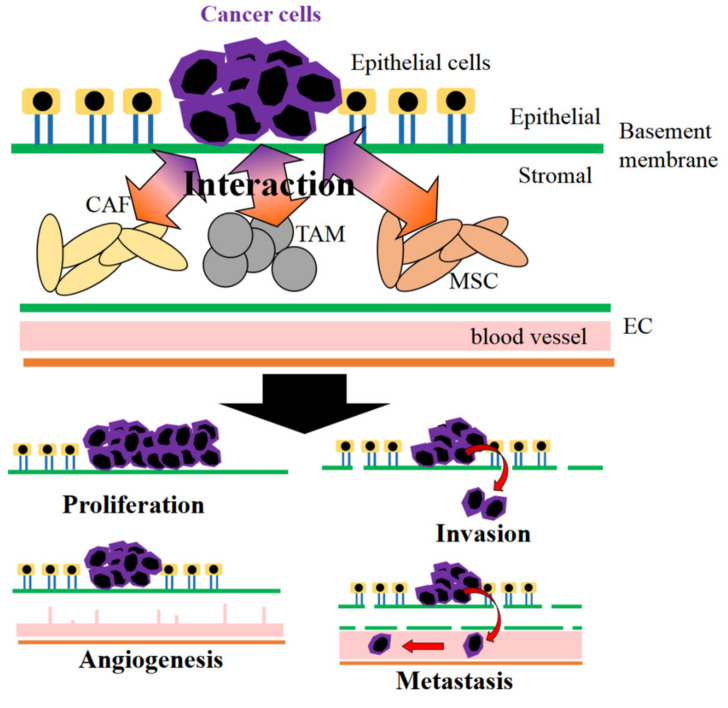
Cancer cells interact with various stromal cells of cancer-associated fibroblasts (CAF), tumor-associated macrophages (TAM), mesenchymal stem cells (MSC), and endothelial cells (EC), leading to the pathological maintenance and promotion of cancer characteristics.

Anticancer drug screening is often performed by using the 2D culture system of cancer cells. As mentioned above, to mimic the cancer environment in the body, the combination of 3D cell culture technology and biomaterials is important. In addition to the technological methods, the interaction of cancer cells with stromal cells should be considered [7], because the cancer environment is composed of several stromal cells, such as cancer-associated fibroblasts (CAF) [8,9], tumor-associated macrophages (TAM) [10,11], mesenchymal stem cells (MSC) [12,13] or endothelial cells [14,15]. It has been demonstrated that cancer cells interact with stromal cells, leading to the promotion of cancer diseases [16] (Figure 2). Moreover, several humoral factors secreted from cells are also important to construct the cancer environment [17,18,19]. Therefore, to mimic the cancer environment or cancer diseases in vitro, a coculture system of cancer cells with stromal cells is essential.

Nowadays, to replicate the cancer environment and diseases in vitro, several studies have been reported on 3D cancer models combined with biomaterials. In this review, first, the important stromal cells and their characterization are briefly described. Second, we introduce 3D cancer models by making use of several biomaterials. 

## 2. Stromal Cells in Cancer Environment

There are four types of stromal cells which are composed of the cancer environment. The biological functions of stromal cells and the humoral factors secreted are briefly explained. Table 2 summarizes some key cytokines in the cancer environment.

### 2.1. Cancer-Associated Fibroblasts

Cancer-associated fibroblasts (CAF) are major stromal cells. CAF of a large-spindle shape are perpetually activated and never undergo apoptosis [8]. Although the origin of CAF is not completely clear, normal fibroblasts [20,21,22], mesenchymal stem cells (MSC) [23,24], or endothelial cells [25,26] are potential sources of CAF. As CAF markers, alpha-smooth muscle actin (α-SMA), fibroblast activation protein (FAP), and fibroblast specific protein-1 are well known [27]. In particular, approximately 90% of cancer cell types show the expression of FAP [28]. The interaction between cancer cells and CAF plays a key role in cancer diseases. An experimental trial to indicate the importance of CAF has been reported by Weinberg et al. Human CAF and breast cancer cells are injected to nude mice. It is demonstrated that cancer cells with CAF effectively proliferate compared with CAF-free cancer cells or cancer cells cocultured with normal fibroblasts groups. This proliferation enhancement was induced by stromal cell-derived factor-1 (SDF-1) secreted [29]. This study clearly indicates the importance of CAF existence for cancer cell activity. CAF not only promote cancer proliferation but also increase the invasion of cancer cells via the cancer–CAF interaction. The interaction also promotes the secretion of various matrix-degrading proteinases. Among them, matrix-metalloproteinase (MMP) has a key role in the cancer invasion or metastasis. MMP can degrade type IV collagen and laminin, which are major components of basement membrane [30,31,32]. In addition to SDF-1 and MMP, transforming growth factor-β1 (TGF-β1) [33,34] and interleukin (IL)-6 [35] are also important factors for the cancer–CAF interaction.

### 2.2. Tumor-Associated Macrophages

Macrophages are usually polarized to M1 or M2 phenotypes responding to the environment. M1 macrophages (proinflammatory) have a capacity of inflammation induction, chronic inflammation, and pathogen defense [36,37]. On the other hand, M2 macrophages (anti-inflammatory) are involved in noninflammatory response, wound healing, and tissue regeneration [37,38,39]. TAM are generally recognized as M2-type macrophages [40,41]. Due to the M2-type phenotype, CD163 and CD204 are well known as the TAM markers [42,43]. The stimulation of macrophages by lipopolysaccharide (LPS) and adenosines can induce TAM in vitro [44]. TAM play an important role in cancer progression. Grivennikov et al. indicate that IL-23 and IL-17 secreted from TAM promote the cancer proliferation [45]. Tumor-necrosis factor-α (TNF-α), vascular endothelial growth factor (VEGF), and TGF-β1 secreted from TAM can promote the cancer metastasis [46]. Taken together, TAM are recognized as important cells for cancer diseases. This promising TAM-targeted therapy has been investigated [47,48].

### 2.3. Cancer-Associated Fibroblasts and Tumor-Associated Macrophages for Different Cancer Types

CAF and TAM are major components of stromal cells in the cancer environment. However, their biological contribution and influence on cancer cells generally depend on the cancer regions. For example, in brain, liver, or kidney cancer, contribution of TAM is larger than that of CAF, while the effect of CAF on the lung or pancreatic cancer is high compared with that of TAM. This is mainly because of the existence ratio [49]. Therefore, the CAF/TAM contribution ratio should be considered to understand the characteristics of various cancer cell types. 

### 2.4. Mesenchymal Stem Cells

Mesenchymal stem cells (MSC) have been noted in the field of tissue regeneration because MSC have a capacity of differentiation into bone, cartilage, or fat cells [50,51,52]. Therefore, MSC transplantation would be effective in regenerative medicine [53]. However, the differentiation capacity of MSC is unfavorable for cancer patients. For example, TGF-β1 secreted from several cells in the cancer environment can differentiate MSC into CAF [54]. Chowdhury et al. also report that exosomes secreted from cancer cells promote the differentiation MSC into CAF [55]. In addition to the differentiation into CAF, MSC also allow TAM to migrate into the cancer environment via C-C chemokine receptor type 2 (CCR2) [56]. Moreover, IL-6 and angiopoietin-1 secreted from primary human MSC can promote the angiogenesis [57]. Recently, it has been reported that MSC can polarize into a proinflammatory MSC-1 and an immunosuppressive MSC-2 phenotype. MSC-2 can enhance the cancer proliferation, spread, and promotion, while MSC-1 suppress the cancer proliferation [13,58,59]. The understanding of MSC roles at cancer sites would provide an important aspect for further cancer research and therapies.

### 2.5. Endothelial Cells

It is important for cancer cells to induce angiogenesis in terms of nutrient and oxygen supply, the elimination of waste products, invasion, and metastasis. However, since a vascularization suddenly advances at the cancer sites under a nonphysiological condition, it is well recognized that the blood vessels in the cancer environment are fragile and the wall is highly permeable. Enhanced permeation and retention effect (EPR effect) is a concept to symbolize this condition of cancer blood vessels [60]. Based on the EPR effect concept, a positive targeting of micelles containing anticancer drug to cancer has been reported [61,62]. Thus, there are some structural and functional differences between the cancer and normal blood vessels. To study cancer characteristics or therapeutic efficacy, the blood vessel properties and the cancer–endothelial cell interaction are important to consider. Some research has been reported to demonstrate that tumor endothelial cells (TEC) differ from normal endothelial cells in properties, such as the cell proliferation, the gene expression, the response to growth factors, or migration [63,64]. High metastatic tumor-derived TEC (HM-TEC) and low metastatic tumor-derived TEC (LM-TEC) can be isolated from mice. It is demonstrated that the secretion levels of VEGF, MMP-2, MMP-9, and SDF-1 from HM-TEC are higher than from that of LM-TEC [60,65]. It is reported that coculture with endothelial cells facilitates the in vitro culture of cancer cells [66]. 

## 3. 3D Culture System of Cancer Cells with Biomaterials

Biomaterials classify into natural biomaterials derived from animals or plants and synthetic biomaterials artificially prepared. Natural biomaterials are composed of polysaccharide (amylose, cellulose, alginate, chitosan, or hyaluronic acid), peptide (collagen or gelatin), nucleic acid, or polyhydroxyalkanoates. Since the degradative enzyme and metabolic system have already existed in the body, most natural biomaterials can enzymatically be degraded. Because the components constitute the cancer environment as the ECM and contribute to cancer diseases, natural biomaterials are often used to design the 3D culture system of cancer cells. Although natural biomaterials are of high biocompatible, there are some limitations of immunogenicity or homogeneity to use. To avoid the issues, synthetic biomaterials are used. Synthetic biomaterials are mainly degraded nonenzymatically based on simple hydrolysis. There are some merits of synthetic biomaterials, such as the characteristics control, the high stiffness, and the clarity of properties. 

In this chapter, several 3D culture systems of cancer cells combined with biomaterials are introduced. To date, two types of biomaterials have been applied to the 3D culture system of cancer cells. One is the culture system of cancer cells with the biomaterials of a spherical shape. When incubated with microspheric hydrogels of biomaterial, cancer cells naturally form a cell aggregate of a tissue-like 3D structure, which mimics the cancer environment. The disadvantages of this system are the difficulty of cells separation from the cell-hydrogel aggregates, and consequently, the result is often of low repeatability. The other is the culture system of cancer cells with the biomaterials of nonspherical type, such as sponge shapes or nonwoven fabrics. In this system, cells effectively proliferate and migrate on the scaffold. This is suitable for immunohistochemical analysis. Table 3 summarizes the 3D culture systems of cancer cells combined with various types of biomaterials.

### 3.1. Chitosan

Chitosan of poly (1, 4 D-glucosamine), a partially deacetylated derivative of chitin, is a natural cationic linear polysaccharide [127]. Chitin is known as primary structural polymers in arthropod exoskeletons. The antigenic response of chitosan is rather low among organonitrogen compounds, and the stiffness is also enough for the cell scaffold. Therefore, chitosan is used as a blood anticoagulant [128], a wound healing accelerator [129], and a surgical suture [130] and also for cardiac [131], neural [132], bone [133], or vein endothelial [134] tissue engineering. Chitosan is also an effective biomaterial for 3D culture of cancer cells because glycosaminoglycan (GAG), closely to the structure of chitosan, is one major component of ECM in the cancer environment [135]. A chitosan scaffold is reported for the 3D culture system of cancer cells. When human breast MCF-7 cancer cells were cultured on the chitosan scaffold, the cell attachment and proliferation were superior to the regular culture of plastic dish [67].

### 3.2. Alginate

Alginate, purified from seaweed, is a naturally-occurring anionic polysaccharide composed of α-l-guluronic acid and β-d-mannuronic acid [136]. As a pharmaceutical application, sodium alginate has already been used for the treatment of peptic ulcer [137]. One of the alginate merits is the quick gelation or cell encapsulation by ionic crosslinking using divalent metal ions of calcium or ferric ions [138,139]. Second, alginate is thermally stable [140]. The molecular structure of alginate is similar to that of polysaccharide in vivo [141]. Therefore, for the 3D culture system of cancer cells, there are many studies on the encapsulation of cancer cells by using alginate gels. Liu et al. prepare alginate gels to encapsulate head and neck squamous carcinoma cells. In addition, three types of gels with different stiffness are prepared by changing the alginate concentration. It is found that the tumorigenicity, the metastatic ability, and the drug resistance increased at the moderate stiffness [82]. The system is also applied to not only neck squamous cell carcinoma but also the hepatocellular carcinoma reaction [80]. In addition, it is reported that IL-8, inflammatory cytokines, secreted from cancer cells cultured within alginate gels under the hypoxia, was high compared with in 2D culture system [84]. Alginate is widely used as a material of cell encapsulation or scaffold for the 3D culture system of cancer cells.

### 3.3. Collagen

Collagen is the main protein of most tissues and contributes to the physical support of tissues [142]. Therefore, collagen is widely used as a material for nerve [143,144,145], bone [146,147,148], cartilage [149,150,151,152], tendon [153], ligament [154,155], or skin [156,157] tissue engineering. Chen et al. report that the expression of proangiogenic growth factors and the transcript of MMP of human breast MCF-7 cancer cells cultured on collagen sponges increased [158]. For the 3D cancer cell culture, collagen is often used to evaluate the invasion ability of breast cancer cells. This may be mainly because it has been reported that breast cancer cells prefer to migrate into collagen I [86]. When high-invasive breast MDA-MB-231 cancer cells were cultured on a collagen scaffold, the migration ability increased via the epithelial–mesenchymal transition (EMT) [88]. For the bone metastasis models, Bersini et al. prepared collagen hydrogels containing osteoblasts cells on a microfluidic device. Human breast MDA-MB-231 cancer cells were invaded into the collagen hydrogels embedding osteoblasts cells effectively via the CXCL5/CXCR2 system compared with the collagen hydrogel without cells [90]. It is demonstrated that the migration ability of breast cancer cells was induced by the degree of collagen fiber alignment or the fibril bending stiffness of the collagen matrix [87].

### 3.4. Hyaluronic Acid

Mucopolysaccharide, namely GAG, repeating units of amino acid and uronic acid, is a major ECM component in connective, epithelial, and neural tissues. Hyaluronic acid (HA) is a GAG family and is composed of D-glucuronic acid and D-N-acetylglucosamine [159,160]. The advantageous characteristic of HA is recognized by the CD44 surface receptor [161]. The interaction between HA and cells via the CD44 receptor affects the cell functions [162]. For cancer, the HA-CD44 interaction leads to the cancer invasion [163], MMP-2 secretion [164], RhoGTPase activation or c-Src phosphorylation [165], and the expression of TGF-β1 and basic fibroblast growth factor (b-FGF) [166]. Moreover, HA affects the stemness maintenance of cancer cells, leading to tumorigenesis, EMT, or drug resistance because CD44 is a major surface marker for stem cells [167,168]. It has been demonstrated that the higher expression of HA in the cancer environment increased the cancer progression, leading to the poor mortality rate [169]. In addition, the molecular weight of HA is also one of the most important factors for cell response. Rayahin et al. report that the molecular weight of HA affects the macrophage phenotypes. At a low molecular weight (5 kDa), the secretion of TNF-α and nitrite production increased. HA of high molecular weight (3 MDa) enhanced the alginase activity which is the characteristic of M2-type macrophages [170]. Therefore, when HA is selected for a 3D cell culture system, the molecular weight of HA should be sufficiently considered because macrophage phenotypes affect the characterization of cancer cells. David et al. report a 3D culture system of cancer cells by use of HA hydrogels crosslinked with adipic dihydrazide to evaluate the invasion ability of several cancer cell lines [103]. It is found by the same groups that the drug resistance enhanced on the same culture systems compared with that in the 2D culture [104].

### 3.5. Matrigel

Basement membrane (BM), a thin layer of ECM, is between the epithelial and stromal sites [171] (Figure 2). BM has a major role in tissue integrity, specificity, and separation [172]. The components of BM are collagen type IV, laminin, heparan sulfate proteoglycan, various growth factors, cytokines, and chemokines [173]. Although BM is an essential material for biological research, human BM of physiological integrity cannot be obtained. As an alternative, matrigel, an extract of Engelbreth–Holm–Swarm tumor derived from wild mice, is used in vitro and in vivo [173]. The major component of matrigel is laminin-111, and gelation is formed at 37 °C [174].

Kramer et al. report on the investigation method of human HT1080 fibrosarcoma cells by use of matrigel [114]. After that, matrigel is often used for cancer invasion assay [109,110]. Matrigel enables the evaluation of not only the cancer invasion ability but also morphology. High-invasive MDA-MB-231 breast cancer cells cultured on matrigel grew, forming a star-like appearance (invasive characterization), while near-sphere cell aggregates were formed when low-invasive breast MCF-7 cancer cells were cultured [107]. Nowadays, the Boyden chamber has been developed to widely investigate cancer invasion as a reliable method [175,176,177]. The two chambers are separated via matrigel-coated porous filter. Cancer cells are plated in the upper chamber, while the medium with or without invasion modulators are in the feeder chamber. When the high-invasion cancer cells are plated, the filter is degraded, leading to the migration of cancer cells and their localization on the feeder surface of filter. Cancer cells migrated are easily counted by the trypan blue stain or fluorescence intensity. The merit of this assay is not to take a long time (12–24 h) to evaluate [171]. The Boyden chamber is a powerful tool to evaluate the cancer invasion ability or perform a drug screening.

### 3.6. Poly (Lactic-Co-Glycolic Acid)

Poly (lactic-*co*-glycolic acid) (PLGA) of biodegradable lactic acid (LA) and glycolic acid (GA) copolymers are widely used for biomedical applications [178]. As an example, leuprolide-loaded PLGA microparticles are used for the treatment of breast or prostate cancer. The microparticles realize an extended release of leuprorelin, which enables once every few months [179]. The basic properties of PLGA are usually given by molecular weight and the LA/GA ratio. For example, PLGA7520 indicates a copolymer of 20,000 molecular weight, and 75 wt % PLA and 25 wt % PGA. Both the molecular weight and LA/GA ratio determine the crystallinity or glass transition temperature [180], which enables the control of the size, porosity, or stiffness of PLGA particles or scaffolds easily [178,181,182,183,184].

Due to the easiness of the functional control, PLGA particles or scaffolds are also used for the 3D culture system of cancer cells. Sahoo et al. prepare PLGA scaffolds for the human breast MCF-7 cancer cell line by a solvent evaporation method. Since the PLGA scaffolds are hydrophobic, the difficulty of wetting and swelling in the culture medium is often a problem. The incorporation of poly (vinyl alcohol) (PVA) into the scaffolds enhanced the hydrophilic nature, leading to improved cell adherence and proliferation [116]. Besides breast cancer cells, several PLGA sponges have been prepared for a cell line of human liver Hep3B cancer by changing the LA/GA ratio. The sponges were prepared by a supercritical CO_2_ gas-foaming method. The growth, mitochondrial activity, DNA amounts, hepatic function, and invasion ability of Hep3B cells on the sponges became maximum at the ratio of 85/15 [119]. In addition, PLGA porous microparticles have been prepared for ovarian HO-8910 cancer cell growth [115].

### 3.7. Polyethylene Glycol

Polyethylene glycol (PEG) is widely used for chemical modification in the field of drug delivery system or biomaterials [185]. PEG-based hydrogels are studied for the 3D cell culture system to investigate the migration of human fibrosarcoma HT-1080 cell line [126] or to mimic the prostate cancer environment [125]. PEG scaffolds in a layer-by-layer fashion with tunable stiffness are reported to evaluate the cell mortality [124]. In addition, Yang et al. report that the mouse breast 4T1 cancer cells are encapsulated in inert PEG hydrogels. The PEG hydrogels enabled cancer cells to form tumorspheres and maintain the cancer stemness [120].

## 4. 3D Culture System of Cancer Cells with Combination of Several Biomaterials

Considering unique properties and functions of each biomaterial, different biomaterials are often combined to use for 3D culture system of cancer cells. In this chapter, the 3D culture systems of cancer cells with combined biomaterials are introduced.

### 4.1. Chitosan–Alginate

Chitosan forms insoluble ionic complexes with alginate to improve the mechanical strength or replicate cancer environment [186,187,188]. Chitosan and alginate (CA) hybrid materials are used to create a 3D material with an interconnected and porous structure. The CA materials have a mechanical strength and shape maintenance significantly improved as compared with chitosan only. This is due to the electrostatic interaction between the amine groups of chitosan and the carboxyl groups of alginate [189]. When human liver HepG2 cancer cells were cultured on the CA scaffolds, both the malignancy and drug resistance increased [68]. The CA scaffolds can be applied not only for hepatocellular carcinoma cells, but also for human glioblastoma U-87 MG and U-118 MG cell lines. The expression levels of genes involved in EMT or cancer stem cells were rapidly promoted [71,72].

### 4.2. Chitosan–Hyaluronic Acid

The mixed hydrogel of chitosan and hyaluronic acid (CH) is often used as a nonadhesive material for spheroids formation. The CH has an ability to maintain the stemness of MSC spheroids through the Rho/Rock activation. A short time of spheroid formation and the enlargement of spheroid size were achieved compared with the conventional culture system [190]. When the 3D spheroids of human nonsmall cell lung cancer cells were prepared on the CH membrane, the expression level of EMT marker, the stemness, or the drug resistance increased compared with those of cells in the 2D culture system [74]. In addition, upon culturing on the CH scaffolds, the expression of stem cell marker and drug resistance of 3D human glioblastoma cancer stem cells was enhanced [70]. A porous CH scaffold promoted the formation of cancer spheroids and their stemness [69].

### 4.3. Matrigel–Collagen or Alginate

Nguyen-Ngoc et al. formulate matrigel hydrogels embedding human breast cancer cell aggregates. Cancer cells are individually dissociated from aggregates to promote their invasion nature because matrigel gives cancer cells a suitable environment. Moreover, the addition of collagen type I into the matrigel increased further cancer invasion [86]. It is reported that the mixed alginate matrigel hydrogel (a mixing ratio of 50:50) enabled human breast cancer cells incorporated to replicate the cancer invasion [79].

### 4.4. Polyethylene Glycol–Other Biomaterials

For the formation of cancer cell scaffolds, PEG is often conjugated with various biomaterials of collagen [91], HA [106], PLGA [118], fibrin [123], and fibrinogen [121,122,185]. PEG/collagen hydrogels of interpenetrating network are prepared to investigate the functions of human breast cancer cells, such as their proliferation, viability, or migration [91]. PEG/HA hydrogels with different stiffness are prepared by changing the PEG concentration to investigate the behavior of brain cancer cells embedded into the hydrogels [106]. Lipke groups have intensively studied the function of cancer cells cultured with PEG/fibrinogen materials [121,122]. Fibrinogen is one of the ECM components and has an important role in the polymerization or deposition of collagen [191]. Breast cancers [121] and colon or prostate cancer cells [122] are embedded in the 3D PEG/fibrinogen hydrogel to experimentally confirm the possibility of a long-time culture. Girard et al. culture several cancer cells on the 3D nanofibers of PLGA-PLA-PEG. Tight irregular aggregates were formed similarly to those of cancers in vivo, and the EMT was induced [118].

## 5. 3D Coculture System of Cancer and Stromal Cells Combined with Biomaterials

### 5.1. Alginate

Coculture of cancer cells and stromal cells with alginate has been investigated. Alginate hydrogels encapsulating human breast MCF-7 cancer cell aggregates were cocultured with human fibroblasts. The oestrogen receptor and the membrane E-cadherin expression increased, the polarity was lost, and the cell migration and angiogenesis increased, in contrast to the monoculture of MCF-7 cells [78]. These phenotypic alterations are important at the advanced stage of cancer. Liu et al. embed hepatocellular carcinoma in the algiante hydrogels, and then, the hydrogels are cocultured with MSC. In this culture system, efficient induction of EMT and the metastasis of cancer cells via TGF-β were observed [81].

### 5.2. Collagen

Nikkhah groups prepare a 3D microengineered cancer model composed of breast cancer cells and CAF embedded into collagen hydrogels. This culture system enabled cancer cells and CAF to achieve their interaction in vitro, which leads to better evaluation of invasion level of cancer cells, MMP secretion, and drug resistance [89]. 3D lung or pancreatic cancer cell aggregates embedded in collagen hydrogels are cocultured with CAF. Cancer cells were attached to CAF and quickly migrated on the CAF protrusions, while CAF-free cancer cells hardly invaded into the matrix [93].

### 5.3. Gelatin

Collagen of one ECM components is often used in the research field of 3D cell culture. However, collagen is water-insoluble and has biological activities, such as blood coagulation and a specific affinity for humoral factors. Considered as a material to design the cell culture system, the inherent properties are sometimes not suitable. Gelatin, a denatured form of collagen, is a cell friendly (high cell adhesion and low inflammation induction) material and is water-soluble [192,193]. In addition, it is technologically easy to prepare gelatin with various physicochemical properties by changing the preparation process from collagen [194,195]. Hydrogel formulations of water-insoluble gelatin can be freely prepared by the physical or chemical crosslinking methods, while the degradation profile can be modified as well [194,196]. The gelatin material is used for a coculture system of cancer cells and stromal cells. Netti groups have extensively investigated cancer microtissues by use of gelatin porous microbeads (GPM). Gelatin scaffolds with interconnected pores of about 20 μm diameter are designed for a 3D culture system, and the microtissues of cancer are formulated [197]. 3D CAF microtissues with GPM showed the higher deposition of collagen, fibronectin, and hyaluronic acid than that of GPM-free 3D CAF. GPM are effective materials to replicate the 3D cancer-stroma condition in vitro [97]. Moreover, human MCF-7 breast cancer and CAF microtissues with GPM are prepared to mimic the cancer microenvironment. The diffusion coefficient of anticancer drugs and the drug action for the 3D MCF-7-CAF microtissues with GPM were higher than those for the GPM-free 3D MCF-7-CAF. In addition, there was a good correlation of the expression of some cancer biomarkers related to cell junctions between the 3D MCF-7-CAF microtissues combined with GPM and in vivo cancer site [98]. The combination of endothelial cells with the culture system is reported [66].

### 5.4. Hyaluronic Acid

As a coculture system of cancer cells and stromal cells with HA, a multilayer system of high-invasive prostate C4–2B cancer cells, or endometrial Ishikawa cancer cells and stromal cells with HA hydrogels is reported. This culture system enables the evaluation of the cytotoxicity of compounds used clinically for both prostate and endometrial cancer cells in vitro. In addition, it is technically possible to anticipate and identify drugs that fail in clinical trials [105]. Han et al. prepare multicellular spheroids of human cell lung carcinoma cell line A549 and human MSC isolated from adipose tissue on CH coating plates. It is found that the gene expression levels of tumorigenicity markers in cancer cells associated with cancer stemness, EMT property, and cell mobility were up-regulated in the MSC-tumor multicellular spheroids [73].

### 5.5. Matrigel

There are several reports on matrigel-assisted coculture systems with stromal cells, such as fibroblasts [108], regulatory T lymphocyte (T_REG_ lymphocyte) or natural killer cells (NK cells) [111], and MSC [112]. Augustine et al. culture both T_REG_ lymphocytes and NK cells with luminal phenotype MCF-7 and basal phenotype MDA-MB-231 to study the immune reaction of breast cancer progression. Cancer morphology, the expression of biomarkers, and CC-chemokine 4 (CCL4) secretion were influenced by the phenotype of breast cancer cells and their immune stimulation [111]. MSC are cocultured with estrogen receptor-positive breast cancer cells embedded in matrigel. Cancer cells rapidly proliferated compared with the MSC-free cells [112].

### 5.6. Collagen–Alginate

Mixed hydrogels of collagen and alginate are investigated to form the multicellular spheroids of human breast cancer cells and fibroblasts. The hydrogel system developed in this study enables the control of the stiffness without altering the major gel components, since the concentration of alginate and collagen in the hydrogel remains constant. The change in the degree of calcium crosslinking does not affect the cell adhesion on the collagen network [85]. Alginate has been extensively used as a material whose stiffness can be readily regulated.

An increase in ECM stiffness is involved in the cancer progression [198]. In addition, there have been reports on the relationship between the stiffness and drug resistance [199,200]. Based on these findings, it is important to design the 3D culture system of cancer cells by making use of biomaterials of which the stiffness can be changed. It has been recently reported that the stiffness of biomaterials affects the characteristics of cancer cells, such as drug resistance [80,201,202,203]. It is promising for the 3D coculture of cancer and stromal cells to use biomaterials of the right material for the right place.

## 6. 3D Coculture System of Cancer and Stromal Cells Combined with Biomaterials of Drug Delivery System

The drug delivery system (DDS) is defined as a technology and methodology to enhance the biological activities of drugs or reduce the adverse effects by appropriately combining with biomaterials. To date, the DDS has been mainly used for in vivo cancer therapy through drug delivery [62,204,205]. However, the technology and methodology are also applicable for drug screening because cancer–environmental normal cell interaction is biologically supported by humoral factors secreted from the cells [8,12,13,16,19,27,33]. The combination of humoral factors in the DDS will enable the enhancement of the interaction between cancer and stromal cells which physiologically takes place in the body.

**Figure 3:**
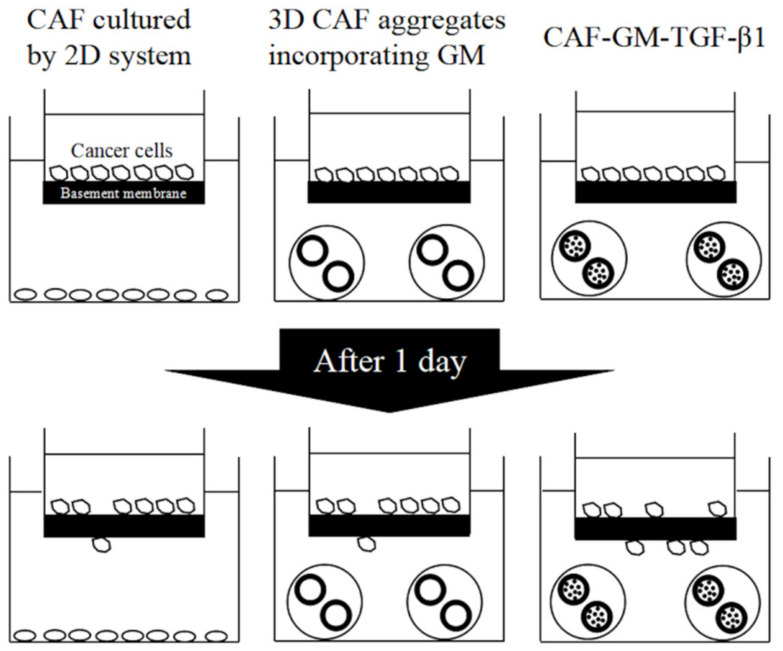
Illustration of cancer invasion based on a combination of 3D cell culture and drug delivery system technology.

Gelatin hydrogel microspheres (GM) for regenerative medicine have been explored. GM can incorporate various growth factors, such as b-FGF [206,207,208,209], TGF-β1 [100,210,211], insulin-like growth factor-1 [212,213], or SDF-1 [214] for controlled release. Growth factors and gelatin molecules effectively interact by physicochemical interaction (e.g., ionic or hydrogen interaction) [194]. Due to the interaction, the mechanism of gelatin matrix-degradation-driven drug release is achievable. This is different from the conventional release system where the drug is usually released from release matrices by the drug diffusion. In addition, GM are in vivo and in vitro enzymatically degraded with time, and finally disappear. The characteristic behavior of GM disappearance is essential as a material for drug release used for tissue regeneration. To repair the damaged tissues, cells should migrate, proliferate, and differentiate. If drug release materials remain for a long time period after drug release is completed, the material remaining will cause the physical impairment of tissue regeneration. The speed of tissue regeneration should be synchronized to that of material degradation. Taken together, the growth factor release as the result of GM degradation with time is effective in realizing tissue regeneration based on the cell activity enhancement for natural healing potential [101,193,215,216,217,218,219]. In addition, a water phase of GM matrices is a pathway to permeate oxygen or nutrients [220]. This permeability is very important considering the 3D cell culture because cells in cell aggregates easily die because of the lack of oxygen or nutrients [221,222,223]. As a trial to break through the issue and culture 3D cell aggregates for a long time period, GM incorporation into the aggregates has been attempted [224,225,226]. Moreover, to enhance the cell activity, drugs to activate the cell function can be impregnated into GM for sustained release. Incorporation of GM containing drugs in cell aggregates is useful to give cells cultured in the 3D system a better condition. It is reported that CAF aggregates incorporating GM containing TGF-β1 (3D CAF-GM-TGF-β1) showed an activated function of CAF. When the activated CAF aggregates and cancer cells were cocultured via a model basement membrane, the invasion rate of cancer cells through the membrane was significantly higher than that of 2D cultured CAF (Figure 3) [100]. The findings indicate that the combination of 3D cell culture and DDS technology is promising to enhance the activity of cancer cells in the 3D culture system. TAM aggregates incorporating GM containing adenosines (3D TAM-GM-adenosines) were formulated to activate and maintain TAM functions. It is found that a 3D cancer cell coculture system of combined 3D CAF-GM-TGF-β1 and 3D TAM-GM-adenosines enabled the effective evaluation of the in vitro invasion of various cancer cells [96].

The body tissue fundamentally consists of cells and the surrounding environment. The environment generally is made of ECM and nutrients for cells. In the case that the two factors of cell environment were not biologically sufficient, the functions of cells would rapidly decrease. The gelatin hydrogel microspheres (GM) function not only as the cell scaffold, but also as the release carrier of TGF-β1 and adenosines of nutrients for CAF and TAM.

## 7. Future Prospective and Conclusion

Biomaterials can assist the 3D culture system of cancer cells through the biological induction of ECM components. Several studies have reported on 3D culture systems by taking advantage of biomaterials. For further development of the 3D culture system of cancer cells, several biomaterials should be combined considering their unique properties and functions. In addition, substantial and close interaction between tissue engineering and the biological research of cancer cells or cancer environment would bring about further development of the 3D cell culture system for anticancer drug screening. In future, patient-derived cancer cells or stromal cells should be combined with biomaterials selected to allow the culture system to approach a more realistic cancer environment. The 3D culture system with biomaterials is a promising tool for cancer research and anticancer drug screening.

## Figures and Tables

**Table 1:** Comparison of cancer cells culture between 2D and 3D systems.

Points Compared	Culture System	
	2D	3D
Cost	Low	High
Cell proliferation	High	Low
Cell differentiation	Low	High
Reproducibility	Good	Poor
In vivo imitation	Limited	Versatile
Cell–cell interaction	Low	High
Cell morphology change	Low	High
Diverse polarity	Loss	Diverse
ECM synthesis	Low	High
Drug sensitivity	High (in contrast to in vivo)	Low (Same as in vivo)

**Table 2:** Cytokines secreted in cancer environment and the biological function.

Cytokines	Functions
Transforming growth factor-β (TGF-β)	Support of cancer cells proliferation Promotion of endothelial–mesenchymal transition (EMT) and the consequent invasion or metastasis Recruitment of fibroblasts Differentiation of fibroblasts or MSC into CAF Promotion of tumorigenicity Promotion of angiogenesis
Tumor necrosis factor-α (TNF-α)	Disruption of epithelial barrier Promotion of inflammatory cell infiltration Stimulation of TGF-β-induced EMT Induction of vascular endothelial growth factors (VEGF) secretion
Vascular endothelial growth factor (VEGF)	Promotion of angiogenesis ECM remodeling Promotion of inflammatory cytokine secretion Formation of tumor endothelial cells
Stromal derived factor-1 (SDF-1)	Promotion of angiogenesis by recruiting endothelial cell precursors Recruitment of MSC Promotion of cancer cells proliferation
Matrix metroproteinase (MMP)	ECM degradation and the consequent angiogenesis, invasion, and metastasis Promotion of tumorigenicity
Interuekin-6 (IL-6)	Stimulation of TGF-β-induced EMT Promotion of cancer cell proliferation Promotion of angiogenesis

**Table 3:** 3D culture system of cancer cells combined with biomaterials.

Biomaterials	Characteristics	Types of Cancer Cells Cultured with Biomaterial Scaffolds of Spherical or Other Shapes		Stromal Cells Cocultured with Cancer Cells
		Spherical ^(a)^	Other (Sponges Shapes or Nonwoven Fabrics) ^(b)^	
Chitosan	Derived from crustacean shells Linear cationic polymer Formation of polyelectrolyte complexes with anionic polymers		Breast cancer [67] Liver cancer [68] Glioblastoma [69,70,71,72] Lung cancer [73,74] Prostate cancer [75,76,77]	MSC [73]
Alginate	Derived from seaweed Water-soluble Crosslinked by ions Easy cell encapsulation Nonadhesive nature to cells Easy stiffness control Thermally stable High water-holding capacity	Breast cancer [78,79] Liver cancer [80,81] Head and neck squamous cell carcinoma [82] Leukemia [83]	Liver cancer [68] Breast cancer [84,85] Glioblastoma [71,72] Prostate cancer [75,76] Oral squamous cell carcinoma [84] Lung cancer [84] Gastric cancer [84]	Fibroblasts [78,85] MSC [81]
Collagen	A major component of ECM Low inflammation High cell adhesion Biodegradability Affinity for integrin receptor	Breast cancer [86]	Breast cancer [85,87,88,89,90,91] Prostate cancer [92] Pancreatic cancer [93] Lung cancer [93,94,95]	CAF [89,93] Macrophages [94,95] Fibroblasts [85,93,94,95]
Gelatin	Denatured material of collagen Water-soluble Crosslinked by chemical or thermal methods Biodegradability High water-holding capacity Affinity for integrin receptor	Breast cancer [66,96,97,98,99] Lung cancer [96,100,101] Liver cancer [96] Pancreatic cancer [102]		CAF [96,97,98,99,100,101,102] TAM [96] Fibroblasts [66,97,102] Endothelial cells [66]
Hyaluronic acid	A major component of ECM Water-soluble Affinity for CD44 receptor High water-holding capacity High molecular weight affects the biological functions.		Glioblastoma [69,70,103] Lung cancer [73,74,104] Gastric cancer [103,104] Prostate cancer [103,105] Osteosarcoma [103] Liver cancer [103] Breast cancer [103] Glioblastoma [106] Endometrial adenocarcinoma [105]	MSC [73] Endometrial stromal sarcoma [105]
Matrigel	Alternative material of basement membrane Derived from mouse tumors Layer used for Boyden chamber Suitable for invasion assay	Breast cancer [79,86]	Breast cancer [107,108,109,110,111,112,113] Fibrosarcoma [109,114] Melanoma [109]	Fibroblasts [108,113] T_REG_ lymphocyte [111] NK cells [111] MSC [112] Endothelial cells [113]
Poly (lactic-*co*-glycolic acid)	Porosity morphology Biodegradability Hydrophobic property	Ovarian cancer [115] Breast cancer [116]	Breast cancer [117,118] Prostate cancer [118] Melanoma [118] Ovarian cancer [118] Lung cancer [118] Liver cancer [119]	
Polyethylene glycol	Chemical modification Water-holding capacity	Breast cancer [120,121,122] Lung cancer [123] Prostate cancer [122] Colon cancer [122]	Breast cancer [91,118,124] Lung cancer [118] Melanoma [118] Ovarian cancer [118] Prostate cancer [118,125] Fibrosarcoma [126] Glioblastoma [106]	Fibroblasts [123] Endothelial cells [123]

^(a)^ 3D cell constructs are readily formed; ^(b)^ cells well proliferate and migrate on the scaffold.
